# The estimation of post-transplant lymphocele origin using creatine kinase activity

**DOI:** 10.3109/03009731003793122

**Published:** 2010-07-19

**Authors:** Jaroslav Pacovsky, Radomir Hyspler, Pavel Navratil, Alena Ticha, Milos Brodak

**Affiliations:** ^1^Department of Urology—Regional Transplant Center, University Hospital and Charles University Medical School, Hradec KraloveCzech Republic; ^2^Department of Gerontology and Metabolism, University Hospital and Charles University Medical School, Hradec KraloveCzech Republic

**Keywords:** Creatine kinase, glutamyl transpeptidase, post-transplant lymphocele

## Abstract

**Introduction:**

The aim of this research was to create a laboratory instrument for the estimation of post-transplant lymphocele origin. It is based on the enzymatic activity of creatine kinase (CK) in the lymphocele content.

**Material and methods:**

A total of 120 lymph samples from different retroperitoneal regions were obtained from non-transplanted patients; equal numbers from the iliac region, renal cysts, and the subdiaphragmatic retroperitoneum. Activities of creatine kinase (CK) and γ-glutamyl transpeptidase (GGT) were determined in all samples and statistically analyzed against their activity in serum from patients without surgery.

**Results:**

Activities of CK in the pelvis, retroperitoneum, renal cysts, and serum were 5.06, 0.83, 6.48 (*P* < 0.001), 2.50, 0.73, 3.60 (*P* < 0.001), 0.02, 0.01, 0.05 (*P* < 0.001), and 0.66, 0.41, 0.79 μkat/l, respectively. Activities of GGT in the same lymph samples were 0.26, 0.16, 0.36 (*P* = 0.048), 0.41, 0.25, 0.48 (non-significant), 0.11, 0.07, 1.17 (*P* = 0.003) and 0.34, 0.24, 0.55 μkat/l, respectively. A graph was constructed relating CK activity to lymph origin.

**Conclusion:**

Significantly different CK enzyme activity was observed in different regions of the retroperitoneum. The presented graph is a simple instrument for the estimation of the lymphocele content origin. The method requires percutaneous aspiration of the lymphocele and evaluation of the CK and GGT activity in the sample. From the graph the estimated proportion of renal lymph in the lymphocele can be read directly. This instrument can provide better understanding of post-transplant lymphocele fluid source.

## Introduction

Lymphocele is a pseudocystic entity with lymph content covered with a hard fibrous capsule. It can be a complication of any surgery involving the lymphatic system. The first lymphocele following a lymphadenectomy together with radical hysterectomy due to cancer was described in 1958 (1,2). Lymphocele can be found in the postoperative period following pelvic or retroperitoneal lymphadenectomy in patients with urologic or gynecologic carcinoma. Experiences with lymphocele complicating the post-transplant course have appeared in the literature since 1969 (3). Hand in hand with kidney transplantation progress, post-transplant lymphoceles became the most common type of lymphoceles.

The incidence generally varies between 12% and 20% (4,5). Post-transplant lymphocele most probably develops from lymph released from injured lymph vessels and is enclosed within a fibrous wall which is formed later. The detailed stages of lymphocele pathogenesis are still unclear.

The lymph fluid inside a lymphocele can originate from the transplanted kidney or from the recipient's iliac vessels. The kidney has three lymphatic vascular systems: parapelvic, cortical, and the extrarenal system of the fatty capsule. All of them run down into the renal sinus and continue along the renal blood vessels as renal lymphatic vessels. The majority of them are transected or opened at the time of organ-procurement surgery or later during ‘back table’ work. If they are not clipped or sutured, they remain open and become an important source of free retroperitoneal lymph—the basis of the future lymphocele. A similar situation can be found during preparation of the recipient's iliac blood vessels. Lymphatic vessels run along the iliac blood vessels. In cases of careless surgery, these fragile lymphatic tissues can be destroyed and provide another lymph source for post-transplant lymphocele.

Identification of the true source of the lymph inside the lymphocele would seem to be important for understanding the pathogenesis. Unfortunately, till now, we have had no effective means of such identification. There is a wide range of visual approaches for diagnosis of lymphocele, and some of them could be expected to help locate the lymphocele origin (6,7). The most promising has been lymphography, but its sensitivity is too low and is thus not suitable for clinical practice (8).

The aim of our study was to determine lymphocele origin by enzymatic analysis of its content. The background of this work is the observation that enzymatic activity in the lymph often corresponds to the enzymatic activity in its tissue of origin. In the case of post-transplant lymphocele it is necessary to use enzymes with high activity in the kidney and in the iliac lymph. We have chosen the enzymes creatine kinase (CK) and γ-glutamyl transpeptidase (GGT).

The most significant source of lymph in the iliac lymphatic vessels is the striated muscles of the lower limbs. CK is an enzyme expressed in various tissues and cell types but predominantly in striated muscle. CK catalyzes the reversible conversion of creatine and adenosine triphosphate (ATP) to phosphocreatine and adenosine diphosphate (ADP). Phosphocreatine serves as an energy reservoir for rapid buffering and regeneration of ATP in tissues and cells that consume ATP rapidly, especially skeletal and cardiac muscle. Due to its high muscle specificity CK activity was used as a marker of iliac lymph (9,10).

On the other hand, the transplanted kidney has different enzymatic equipment. The enzyme GGT is a glycoprotein attached to the external surface of various cell types. Its physiological function in glutathione (GSH) metabolism is to recover cysteine from extracellular GSH. High GGT activity has been observed in cells that exhibit intense secretory and absorptive function, such as epithelial cells of the proximal tubuli in the kidneys or of the biliary ducts in the liver. In the kidney, the primary site of GGT activity is the outer surface of the microvillus membrane (brush border) in the proximal tubular cells. It participates in the reabsorbtion of small proteins, peptides, and amino acids from the proximal tubule of the nephron. The kidney and liver are the organs with the highest activity of GGT (11–15), and GGT activity was hence used as a marker of renal lymph.

Despite the growth in kidney transplantation, a suitable method for determination of lymphocele origin is still lacking. The objective of the present work was to find a laboratory technique for identification of the lymphocele origin using CK and GGT activity in the lymphocele content.

## Material and methods

**Figure 1.**
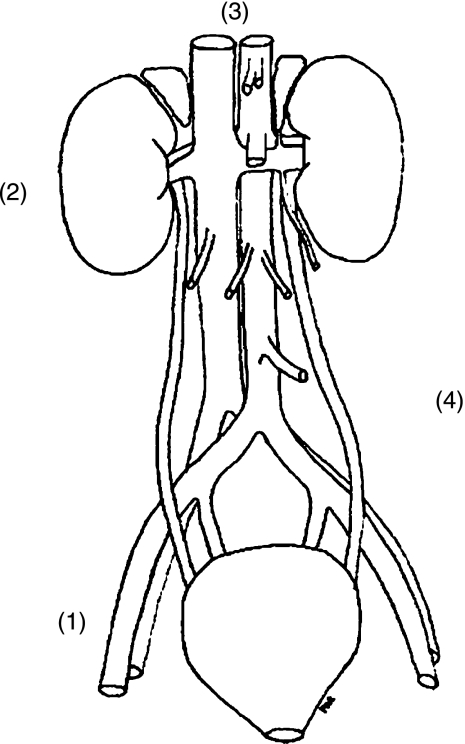
Locality where lymphatic samples were harvested: 1 = iliac lymph, 2 = renal cyst, 3 = subdiaphragmatic space, 4 = blood.

The first objective of this work was mapping of the CK and GGT activity in different regions of the lymphatic system in the retroperitoneal space. Lymph samples were collected from non-transplanted patients who had undergone lymph node surgery in our department for surgically curable malignant disease (prostatic, ovarian, testicular cancer). The patients were equally of both genders, and their age was from 18 to 75 years. The lymph samples were taken from the postoperative drains with prolonged postoperative lymph output for more than 4 days. Forty samples were collected from patients following pelvic lymphadenectomy, representing lymph from the pelvis and lower extremity. A further 40 samples were collected from patients following retroperitoneal lymphadenectomy, representing combined pelvic and renal lymph. The sampling of clear renal lymph is extremely complicated, almost impossible in clinical conditions. For this reason we collected a further 40 samples from aspirated renal cysts (Figure 1).

The activity of CK depends on the status of skeletal muscle, and it is always elevated postoperatively. All these specimens were collected only if the serum CK activity had been normalized. We used 40 serum samples from patients without surgery as a control group. Lymph and serum samples were analyzed for activity of CK and GGT and concentration of total protein using Modular analyzer (Hoffmann La Roche Ltd, Basel, Switzerland) and original reagents.

The total activity of creatine kinase was determined using International Federation of Clinical Chemistry (IFCC) recommended kinetic procedure based on the reversible transfer of the phosphate group from creatine phosphate to Mg-ADP. The resulting Mg-ATP was determined using hexokinase and glucose-6-phosphate dehydrogenase as a rise in hydrogenized nicotinamide adenine dinucleotide phosphate (NADPH) concentration measured spectrophotometrically at 37°C. Some authors identified the reactivation time (the time of reaction mixture incubation with N-acetylcysteine before the reaction initiation) as a critical parameter (16). CK may be partially inactivated in the sample by oxidation of the sulfhydryl groups in the active center, and it is reactivated during incubation time with N-acetylcysteine. This reactivation time used during CK assay was 180 seconds. The coefficient of variation 0.9% was calculated as a measure of analytical precision using certified material (Hoffmann La Roche Ltd, Basel, Switzerland).

The GGT activity was determined during the kinetic procedure using L-γ-glutamyl-3-carboxy-4-nitroanilide as substrate and glycylglycine as acceptor. The amount of 5-amino-2-nitrobenzoate liberated from substrate is proportional to the GGT activity and was measured spectrophotometrically at 37°C. The coefficient of variation 2.76% was found using certified material. The activity of both enzymes was expressed in μkat/l.

During a small pilot study, the lymphocele content aspirated during standard treatment of post-transplant lymphocele was analyzed in six patients (four males, two females), and the proportion of renal lymph was estimated.

All procedures were carried out after signed confirmation of the patient's informed consent and with approval of the Local Ethical Committee according to the Declaration of Helsinki.

Statistical analysis of results was performed using the Wilcoxon signed-rank test, and *P*-value < 0.05 was considered as significant (SigmaStat Software, Systat, USA). Non-commercial software created specifically for this work in the Department of Mathematics of the University of Hradec Kralove was applied for visual modeling of CK and GGT lymphatic activity in the retroperitoneal space.

## Results

**Figure 2.**
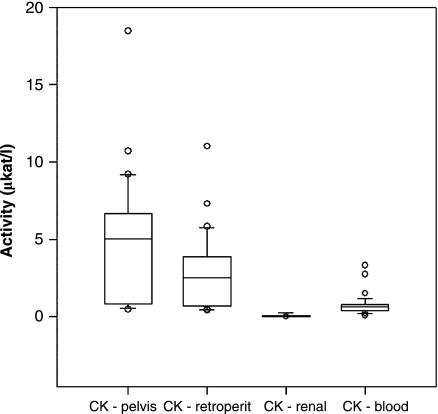
The creatine kinase (CK) activity in lymph from various sources versus serum (pelvis *P* < 0.001, retroperitoneum *P* < 0.001, renal cyst *P* < 0.001).

**Figure 3.**
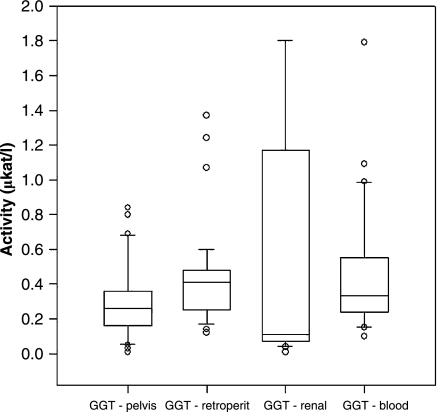
The γ-glutamyl transpeptidase (GGT) activity in lymph from various sources versus serum (pelvis *P* = 0.048, retroperitoneum NS, renal cyst *P* = 0.003).

The activities of CK and GGT enzymes found in different regions of the retroperitoneal space are presented in Figure 2 and Figure 3, respectively. In the case of CK, all sites were significantly different from each other, with the highest activity in pelvic lymph and lowest in renal lymph. No statistically significant differences were found in the case of GGT activities in the lymph and serum samples. Protein concentrations (mean ± SD) of 25.2 ± 9.9 g/l (pelvic lymph), 40.3 ± 6.38 g/l (retroperitoneal lymph), and 49.0 ± 15.6 g/l (renal lymph) were observed, and these values match published results (17).

**Figure 4.**
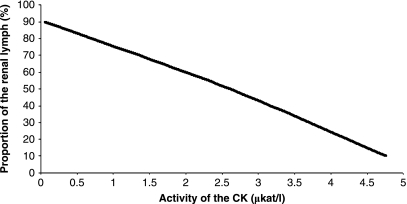
Graph for deduction of renal lymph ratio in the lymphocele, based on creatine kinase (CK) activity in lymphocele content.

The diagnostic graph was created (Figure 4) using CK activity assuming complete pelvic origin in pelvic samples, complete renal origin in renal samples, and equivalent pelvic and renal origin in retroperitoneal samples. The curve is ready for practical deployment, and it enables estimation of lymph origin to the order of 10%. This is considered as satisfactory precision for clinical practice.

The regression equation is:

(1) PRL=−17.14CK+91.99

where PRL = proportion of the renal lymph in the lymphocele (%), and CK = activity of CK in the lymphocele content (μkat/l). Notice that if CK < 0.06, then PRL = 100%, if CK > 4.76, then PRL = 0%.

The results of a pilot study performed as mentioned above are presented in Table I.

**Table I.** The results of a pilot study—analysis of the post-transplant lymphocele content and the estimated proportion of renal lymph in a group of six patients.

Patient no.	Patient age (years) and sex (M/F)	Time from renal transplantation (months)	CK activity in lymph from lymphocele (μkat/l)	Total protein concentration in lymph from lymphocele (g/l)	Estimated proportion of the renal lymph in lymphocele (%)
1	70 M	7	0.25	15.2	88
2	58 F	1	1.07	23.3	73
3	50 M	3	0.15	21.9	89
4	30 F	1	4.36	7.5	16
5	55 M	2	1.75	10.8	62
6	30 M	1	0.23	9.6	88

CK = creatine kinase.

## Discussion

This presented study analyzing the enzymatic activity of lymph and its mapping to different regions of the retroperitoneal space is a pilot study. The highest CK activity was found as expected in the pelvic region. Statistically significant differences were found in various lymphatic regions. On the other hand, the highest GGT activity was expected in renal lymph compared to other tested lymph sources, but this was not found in all cases. Due to the high scatter of results in renal lymph and insignificant differences of activity in different groups of samples, the use of GGT in the proposed methodology was rejected. The overwhelming part of GGT activity in serum originates from the liver and probably produces the scatter mentioned above. Moreover, since the major expression site in kidneys for GGT is the microvillus membrane in the proximal tubular cells, most of renal GGT is released to urine, and renal lymph is not enriched sufficiently with this enzyme.

There are also other difficulties with the interpretation of lymph analysis results. The volume of the lymph varies according to hydration, nutritional status, physical activity, and many other factors. In addition, protein concentration in the lymph varies according to sampling site location and thus cannot be used as a correction factor; consequently enzyme activity must be analyzed as a raw parameter.

The data received from the laboratory analyses of the lymph samples were computerized and are presented in the chart (Figure 4). The chart presents the relationship between CK activity in the lymphocele and proportion of the renal lymph contributing to the lymphocele content. Its utilization requires the knowledge of the CK activity in the lymphatic sample from the lymphocele obtained by percutaneous aspiration, and it is possible to use this graph in the estimation of the post-transplant lymphocele origin as a simple deductible chart. It is ready for easy practical use, and the percentage of renal lymph can be deducted from the *y* co-ordinate. When a lymphocele is found, laboratory confirmation is necessary to differentiate it from an accumulation of urine or blood. An ultrasound-guided fine-needle percutaneous aspiration must be performed and the aspirated fluid subjected to biochemical and microbiological analysis. The CK activity may be evaluated in addition to the standard laboratory markers (urea, creatinine, sodium, potassium, total protein, and albumin), and thus the proposed method does not carry additional invasive testing for patients. The laboratory result can be applied to the graph, and the estimated proportion of renal lymph can be read directly from the curve.

The major advantages of the proposed method are the wide availability of CK activity determination, its low cost, and the absence of any requirement for additional invasive procedures. Of course, there are obvious limitations. The method was developed using ‘renal tissue liquid’ obtained from cysts, not exactly renal lymph. It is based on enzyme activity without any correction factor, which could compensate for lymph density changes, such as may be due to variation in patient hydration status. Despite these limitations, the pilot study showed reliable results varying from 20% to 100% of renal lymph content, the remainder consisting of iliac lymph.

A new and simple pilot method to analyze easily the lymphocele content was developed. This technique offers the possibility of estimating lymphocele origin and quantifying the proportions of renal and iliac lymph inside the post-transplant lymphocele. It may thus help in guarding against its formation and possibly in reducing the rate of this frequent complication.
